# Vinorelbine plus trastuzumab combination as first-line therapy for HER 2-positive metastatic breast cancer patients: an international phase II trial

**DOI:** 10.1038/sj.bjc.6603351

**Published:** 2006-09-12

**Authors:** A Chan, M Martin, M Untch, M G Gil, V Guillem-Porta, M Wojtukiewicz, P Kellokumpu-Lehtinen, H L Sommer, V Georgoulias, N Battelli, M Pawlicki, D Aubert, T Bourlard, J Gasmi, G Villanova, L Petruzelka

**Affiliations:** 1 Mount Hospital and Royal Perth Hospital, Perth, Australia; 2 Hospital Clinico San Carlos, Madrid, Spain; 3 Klinikum Grosshadern, München, Germany; 4 Instituto Catala d'Oncologia, Hospitalet de Llobregat, Barcelona, Spain; 5 Instituto Valenciano de Oncologia, Valencia, Spain; 6 Oncology Department, Medical University and Regional Cancer Centre, Bialystok, Poland; 7 Medical School, Tampere University Central Hospital, Pikonlinna, Finland; 8 Klinikum Universität München, München, Germany; 9 Peripheral General Hospital Iraklion, Stavrakia and Voutes, Iraklion, Greece; 10 Azienda Ospedaliera Umberto I, Ancona, Italy; 11 Centrum Onkologii W Krakowie, Krakow, Poland; 12 Institut de Recherche Pierre Fabre, Boulogne-Billancourt, France; 13 General Teaching Hospital, Prague, Czech Republic

**Keywords:** trastuzumab, vinorelbine, first-line chemotherapy, HER 2-positive metastatic breast cancer

## Abstract

The aim of this international phase II trial was to determine the efficacy and safety profile of weekly vinorelbine plus trastuzumab as first-line chemotherapy for women with HER 2-overexpressing metastatic breast cancer. Sixty-nine patients with tumours overexpressing HER 2 received vinorelbine: 30 mg m^−2^ week^−1^ and trastuzumab: 4 mg kg^−1^ on day 1 as a loading dose followed by 2 mg kg^−1^ week^−1^ starting on day 8. Sixty-two patients were evaluable for response and 69 patients were evaluable for toxicity. The overall response rate was 62.9%. The median time to response was 8.4 weeks, the median duration of response was 17.5 months, the median progression-free survival was 9.9 months (95% CI, 5.6–12.1) and the one-year progression-free survival was 39.1%. The median survival for all patients was 23.7 months (95% CI, 18.4–32.6). This regimen was safe: grade 3–4 neutropenia were observed over 17.7% of courses in 83.8% of patients, with only two episodes of febrile neutropenia (0.1%) in two patients (2.9%). Only one patient discontinued treatment due to grade 3 symptomatic cardiac dysfunction that resolved with therapy. Vinorelbine plus trastuzumab is one of the most active treatment regimens for patients with HER 2-positive metastatic breast cancer and demonstrates a very favourable safety profile allowing prolonged treatment with long-term survival. This study has been presented in part at the following conferences: The San Antonio Breast Cancer Symposium, San Antonio, TX, USA, 2003; The American Society of Clinical Oncology, Orlando, FL, USA, 2005.

Advances in understanding breast cancer biology have led to the development of targeted anticancer treatment. HER 2/neu gene amplification, identified in 20–30% of breast cancers, is a prognostic marker for poor clinical outcomes (Slamon *et al*, 1987, 1989; Berger *et al*, 1988; Ravdin and Chamness, 1995). Trastuzumab is a humanised monoclonal antibody that targets the extracellular domain of the HER 2 receptor and inhibits the proliferation of human tumour cells overexpressing the HER 2 protein. Trastuzumab has demonstrated antitumour activity in metastatic breast cancer in first-line treatment, or in patients who have tumour progression after chemotherapy (Cobleigh *et al*, 1999; Vogel *et al*, 2002). Preclinical studies indicated synergistic or additive interactions for trastuzumab with different chemotherapy agents (Pegram *et al*, 1999, 2004). Two randomised trials comparing trastuzumab plus chemotherapy to chemotherapy alone demonstrated an improved outcome for patients receiving trastuzumab plus chemotherapy (Slamon *et al*, 2001; Marty *et al*, 2005). However, trastuzumab therapy can be associated with cardiac toxicity (Slamon *et al*, 2001). Retrospective analyses have found that concomitant use with anthracyclines and advanced age are predictive factors of trastuzumab-induced cardiac dysfunction (Seidman *et al*, 2002).

Among chemotherapeutic agents used in the management of metastatic breast cancer, vinorelbine has demonstrated the greatest antitumour synergy with trastuzumab in preclinical models (Pegram *et al*, 1999, 2004). Furthermore, the weekly administration of both agents and the lack of overlapping toxicity between these drugs make this combination suitable for further investigation. Several single institution or national phase II trials have been conducted with this regimen as first-line therapy or in heavily pretreated patients with metastatic breast cancer. These studies consistently demonstrated high efficacy with limited toxicities (Burstein *et al*, 2001, 2003; Jahanzeb *et al*, 2002; Bayo-Calero *et al*, 2004; Bernardo *et al*, 2004).

This international, open-label, phase II study was designed to evaluate the efficacy and safety of this combination as first-line treatment for metastatic breast cancer.

## MATERIALS AND METHODS

### Eligibility criteria

Eligible patients were female >18 years, with histologically proven metastatic breast carcinoma. Other inclusion criteria included Karnofsky performance status (KPS) score ⩾70%, at least one bidimensionally measurable lesion according to the World Health Organisation (WHO) criteria and a life expectancy >16 weeks. Adjuvant chemotherapy containing an anthracycline was allowed if the total cumulative dose of doxorubicin or epirubicin did not exceed 360 and 720 mg m^−2^, respectively. A disease-free interval of more than 6 months between the last dose of adjuvant chemotherapy and documentation of relapse was needed. Patients were required to give written informed consent before study-specific procedures were performed and to be able to comply with the protocol for the duration of the study. Patients were required to have normal cardiac function with left ventricular ejection fraction (LVEF) measured by echocardiography or multigated acquisition scan (MUGA scan) no more than 10% below the normal limit of the institution.

Patients were ineligible if they had local relapse only, prior chemotherapy in the metastatic setting, previous treatment with vinca alkaloid or trastuzumab, grade >2 peripheral neuropathy, serious illness or medical conditions such as cardiac disease, unstable diabetes, uncontrolled hypercalcaemia or active infection. Patients were also excluded if they were pregnant or lactating, had central nervous system or leptomeningeal metastases, had participated in another clinical trial with any investigational drug within 30 days prior to the study inclusion or had a history of another malignancy, except cured basal cell carcinoma of the skin or excised carcinoma *in situ* of the cervix.

Patients were required to have adequate bone marrow (haemoglobin value ⩾10 g dl^−1^, absolute neutrophil count ⩾1.5 × 10^9^ l^−1^ and platelet count ⩾100 × 10^9^ l^−1^), renal (serum creatinine ⩽130 *μ*mol l^−1^) and liver function (serum bilirubin ⩽1.5 × upper normal limit (UNL), serum glutamic–oxalic transaminase (SGOT) or serum glutamic–pyruvic transaminase (SGPT) ⩽2.5 UNL, unless liver involvement).

The HER 2 status of primary or metastatic tumours was assessed by immunohistochemistry (IHC) and fluorescent *in situ* hybridisation (FISH) on formalin-fixed paraffin-embedded tissue. Because of the possibility of discrepancy between local laboratories for the HER 2 testing with IHC, a centralised testing was implemented (Professor Josef Rüschoff, Institute of Pathology, Kassel, Germany). Immunohistochemistry was performed using the DAKO Hercept® Test, and FISH analysis was performed using the PathVysion *HER 2* probe kit Vysis®. Patients with HER 2 staining assessed 3+ by IHC were eligible for the study, while those with IHC 2+ required confirmation by FISH.

### Treatment plan

Treatment was given on a weekly basis (1 cycle=4 weeks of treatment). The initial trastuzumab infusion was administered intravenously at the dose of 4 mg kg^−1^ over 90 min on day 1. The patient remained under medical supervision for 1 h following completion of the first infusion. Subsequent weekly trastuzumab courses were given at 2 mg kg^−1^ over 30 min. The postinfusion observation period for the second infusion was shortened to 30 min and was withheld over subsequent administrations provided no adverse events occurred.

Vinorelbine was administered after trastuzumab infusion at 30 mg m^−2^ weekly over 6–10 min. The first dose of vinorelbine was administered 2 h after the trastuzumab infusion. Patients were to receive at least 8 weeks of treatment (two cycles).

Diphenhydramine and paracetamol premedication were optional. Treatment was continued until disease progression, unacceptable toxicity or patient's refusal to continue.

### Dose modifications

There was no dose adjustment for trastuzumab. The infusion was stopped if the patient developed chills, fever, allergic reaction or any grade 3–4 toxicities. Trastuzumab administration was also withheld in the event of a decline in LVEF value either ⩾20% of absolute units from baseline to a value above the lower normal limit for the centre, or ⩾10% of absolute units to a value below the lower normal limit for the centre. Trastuzumab therapy was reintroduced 1 month later if there was no further decline in LVEF. Trastuzumab was permanently interrupted if New York Heart Association (NYHA) class III/IV cardiac function impairment occurred.

Vinorelbine was postponed for 1 week in case of grade ⩾3 neutropenia. Following one delay of vinorelbine, the dose of vinorelbine was permanently decreased to 25 mg m^−2^ week^−1^ for the remainder of study treatment. In the event of grade >2 peripheral neuropathy, vinorelbine was permanently discontinued. In case of increased SGOT/SGPT to 5.1–20.0 UNL, or bilirubin at 1.5–3.0 UNL, vinorelbine administration was postponed for 1 week and permanently discontinued in case of three consecutive delays or SGOT/SGPT value >20.0 UNL and/or total bilirubin value >3 UNL. Patients who experienced grade 3 or 4 neutropenia with or without fever were allowed to receive a granulocyte colony-stimulating factor in the subsequent cycles at the investigator's discretion.

### Study assessments

Screening assessments were performed at baseline in all patients within 28 days before study treatment, including medical history, physical examination, performance status, HER 2 testing, electrocardiogram, LVEF, chest X-ray, tumour measurements by computed tomography (CT), abdominal ultrasound, bone scan and brain CT scan. On day 1 of each 4-week cycle, physical examination and performance status were assessed and haematological and blood chemistry measurements were carried out. Additional radiological imaging was performed as clinically indicated.

Complete blood cells counts were performed weekly within 2 days before vinorelbine administration. LVEF measurement was repeated every 6 months and at any time if clinically indicated.

Objective responses were assessed every 8 weeks until progression, or less than 8 weeks if early progression was suspected. The best overall response achieved, using the standard WHO criteria of tumour response, was reported for each patient. A complete response (CR) required complete disappearance of all previously detectable disease with no appearance of new lesions, and partial response (PR) required at least a 50% decrease of the sum of the products of the two greatest perpendicular diameters of all measurable lesions, with no appearance of new lesions or progression of any lesion. Both CR and PR had to be confirmed 4 weeks later. Stable disease (SD) was defined as less than 50% decrease or a less than 25% increase in the size of one or more measurable lesions. Progressive disease was defined as 25% or more increase of previously described lesions, or appearance of new lesions. An Independent Review Panel confirmed all responses. Clinical benefit was defined as patients achieving CR, PR or SD maintained for a minimum of 6 months.

Adverse events and medical history were recorded throughout the study. The WHO criteria were used to grade the adverse events.

### Statistical analysis

The one-sample multiple testing procedure for phase II clinical trials described by Fleming was used. Based on a type I error rate of 5% and a 95% power to reject the null hypothesis of a 20% objective response rate (complete or partial), a sample size of 60 evaluable patients was needed.

All registered patients were included in the intent-to-treat (ITT) analysis; patients who received at least one administration of study treatment were analysed for safety. The objective response was the main end point for this study. The Kaplan–Meier method was applied on overall survival, progression-free survival, duration of response and time to treatment failure.

## RESULTS

### Patients' characteristics

Between October 2000 and June 2002, 69 patients with metastatic breast cancer screened for HER 2 status and fulfilling the eligibility criteria were enrolled in the study at 20 sites from 13 countries. Seven out of 69 patients were not evaluable for response, but were included in the ITT analysis: two patients were not assessable as a result of premature study discontinuation (one patient withdrew from the study after the first therapy administration due to an adverse event, the second one died during the first cycle from sepsis), one patient had bone metastases only which was not assessable, another patient had no measurable disease as defined in the protocol and three other patients were not eligible due to relapse within 6 months from early-stage chemotherapy. Therefore, 62 patients with measurable disease were assessable for disease response as per protocol.

The patient's characteristics are described in Table 1. Median age was 53 years (range 30–74 years), more than 70% of patients had KPS ⩾90% and the median time between initial diagnosis and relapse was 17.8 months. Nearly all patients (97.1%) had tumour HER 2 status 3+ determined by IHC, while two patients required confirmation with FISH. The majority of patients had visceral metastasis (75.4%). Sixty-five per cent of patients had prior neo/adjuvant chemotherapy including anthracycline in the majority of cases.

### Clinical efficacy

Independent review panel observed an objective response rate of 62.9% among the 62 evaluable patients (95% CI, 49.7–74.8), including 14.5% of CRs. Stable disease was observed in 19.4% of patients. The clinical benefit (CR+PR+SD ⩾6 months) was 72.6% (95% CI, 61.5–83.7) Table 2. In the ITT population, the objective response rate was of 58% (95% CI, 45.5–69.8).

**Figure 1:**
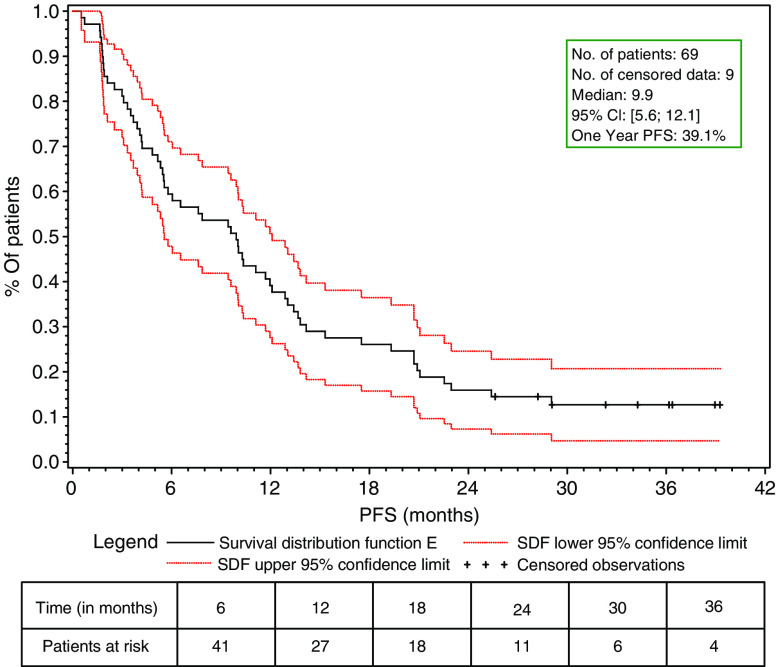
Progression-free survival – ITT population.

**Figure 2:**
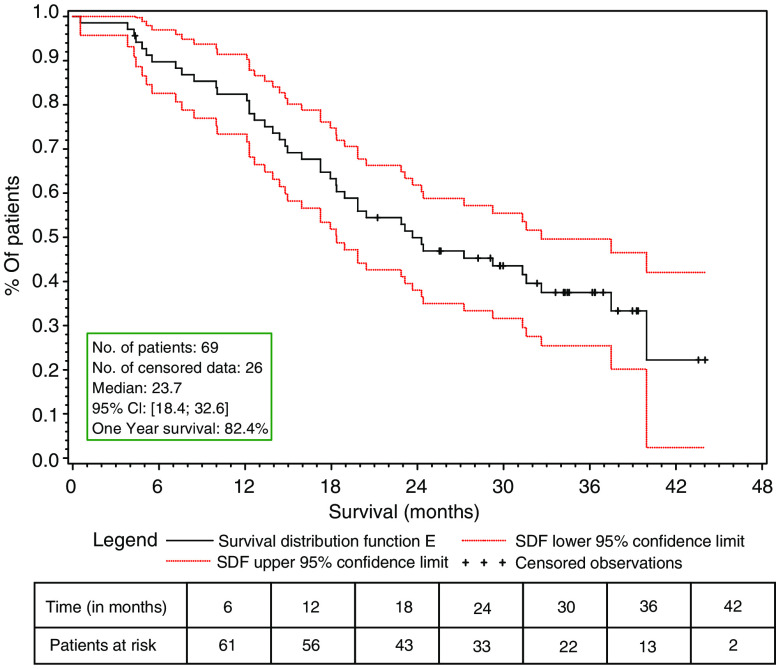
Overall survival – ITT population.

Patients who had received prior adjuvant chemotherapy with anthracyclines or anthracyclines and taxanes had similar objective response rates (61.9 and 54.5%, respectively), as well as those with or without visceral involvement (63.8 and 60% of objective response rates, respectively) Table 2. Patients anticipated to have a worse prognosis, with a disease-free interval of less than 12 months, had an overall objective response rate of 71.4%. The median follow-up of patients was 36.2 months. The median time to response was 8.4 weeks (7.1–31.3 weeks) and the median duration of response was 17.5 months (95% CI, 12.1–23.0). The time to treatment failure was 6 months (95% CI, 5.3–8.6), the progression-free survival was 9.9 months (95% CI, 5.6–12.1) and the median overall survival for all patients was 23.7 months (95% CI, 18.4–32.6) (Table 3; Figures 1 and 2).

Twenty-six patients received further radiotherapy, 24 patients received hormonotherapy and 39 patients received further chemotherapy.

### Dose modifications/reductions

A total of 1769 administrations of vinorelbine and 2242 administrations of trastuzumab were given, with a median number of 18 (range, 1–106) and 24 (range, 1–124) administrations per patient for vinorelbine and trastuzumab, respectively. Fourteen patients received this regimen for more than 1 year, and four patients for more than 2 years.

The median relative dose intensity for vinorelbine was 66.1%, whereas that for trastuzumab was 95%. Vinorelbine treatment was delayed more than 3 days or cancelled in 26.5% of administrations mainly due to haematological toxicity, whereas trastuzumab treatment was delayed more than 3 days or cancelled in 6.7% of administrations mainly due to investigator's decision or patient's convenience.

### Treatment-related toxicity

The most frequent haematological toxicity (WHO grading) was neutropenia, with grade 3–4 neutropenia observed in 83.8% of patients over 301 (17.7%) administrations. Neutropenia was usually brief, infrequently associated with infections and was not cumulative (data not shown). There were only two episodes (0.1%) of febrile neutropenia in two patients (2.9%). One of these patients died from neutropenic sepsis. Grade 3 anaemia was reported in only two (2.9%) patients over three (0.2%) courses. No grade 2, 3 or 4 thrombocytopenia was reported (Table 4).

Nonhaematological toxicities were mild. The most common nonhaematological toxicity (WHO grade 3/4) attributed to study treatment was grade 3 asthenia in six patients (8.8%) and infection in four patients (5.9%). Other side effects were rare: grade 3 peripheral neuropathy and constipation were noticed in two patients each (2.9%) (Table 5).

### Cardiac toxicity

**Figure 3:**
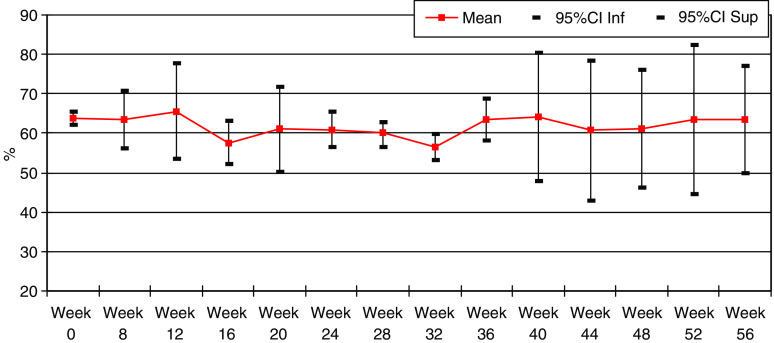
Mean LVEF during treatment.

Eligible patients received study treatment with LVEF no more than 10% below the normal limit of the institution. The mean LVEF at baseline was 63.8% (95% CI, 62.1–65.6), and the minimum value during treatment remained similar at a mean of 59.1% (95% CI, 55.9–61.7) (Figure 3).

One patient experienced a decline of LVEF to 38% with symptomatic cardiac failure 4 months after study initiation. She was aged 68 years old with a history of hypertension. Vinorelbine administration was postponed for 2 weeks and trastuzumab treatment was permanently discontinued. Following initiation of antifailure treatment, the symptoms resolved with the LVEF normalising at 58%. Three other patients experienced asymptomatic decline in LVEF to 41, 46 and 44%, respectively. The therapy was interrupted for 4 weeks and only reintroduced after LVEF increased to ⩾50%.

## DISCUSSION

Trastuzumab combined with chemotherapy is the standard of care for women with HER 2-overexpressing metastatic breast cancer. However, the optimal chemotherapeutic agent to combine with trastuzumab is not established. Ideally, chemotherapy–trastuzumab combinations should produce durable response rates with minimal toxicity. In particular, the known cardiac toxicity of trastuzumab would preclude its use with an agent also known to be cardiotoxic. Data from preclinical models have demonstrated synergistic interaction between trastuzumab and vinorelbine (Pegram *et al*, 2004). Several phase II studies have been conducted in the first-line setting or after progression to other chemotherapy. This doublet has produced consistent response rates ranging from 60 to 86%, with a satisfactory tolerance profile (Burstein *et al*, 2001, 2003; Jahanzeb *et al*, 2002; Bernardo *et al*, 2004; Bayo-Calero *et al*, 2004).

The current study evaluated the combination of vinorelbine (30 mg m^−2^ week^−1^) and trastuzumab (4 mg kg^−1^ on day 1 followed by 2 mg kg^−1^ week^−1^ starting on day 8) as first-line therapy for HER 2-positive metastatic breast cancer patients. This is the first international phase II trial with centralised HER 2 testing evaluating this combination.

The short disease-free interval of 17.8 months and prior use of adjuvant anthracyclines with or without taxane chemotherapy in 52.2% of patients underlies the aggressive nature of disease in this population under study. The clinical benefit of 72.6% with a median duration of response of 17.5 months compares favourably with other trastuzumab combinations (Seidman *et al*, 2001; Esteva *et al*, 2002; Marty *et al*, 2005). The duration of efficacy of the current combination is reflected by the 39.1% disease-free survival rate at 1 year. By 9 November 2005, two patients were still on treatment with vinorelbine and trastuzumab after 3.5–3.6 years, respectively.

This multicentre study confirms the efficacy of vinorelbine with trastuzumab previously seen in other studies, with independent radiological review avoiding investigator assessment bias. Burstein reported 75% of response rates without grade 3–4 cardiac toxicity in a single centre trial of weekly vinorelbine (25 mg m^−2^) and trastuzumab (4 mg kg^−1^ on day 1 then 2 mg kg^−1^ week^−1^) used as first- and second-line therapy (Burstein *et al*, 2001). In a multi-institutional trial of the same schedule, the same author confirmed the efficacy of trastuzumab plus vinorelbine as first-line chemotherapy, with 68% of patients achieving an objective response and 39% of patients remaining free of disease progression at 1 year (Burstein *et al*, 2003). Jahanzeb *et al* (2002), in a trial of first-line vinorelbine and trastuzumab at the same schedule and doses as the current study, reported 78% of responses among 40 patients. In addition, the time to disease progression was extended to 17 months.

The results are in line with other reports using different chemotherapies in combination with trastuzumab. The objective response rate was of 50% in the study by Slamon *et al* (2001) with the combination of trastuzumab and paclitaxel and 61% in the study by Marty *et al* (2005) with trastuzumab plus docetaxel.

The toxicities associated with vinorelbine and trastuzumab were predictable and manageable. Although grade 3 or 4 neutropenia occurred in 83.8% of patients, only two episodes (0.1%) of febrile neutropenia occurred (2.9% of patients).

Cardiomyopathy, a serious toxicity associated with trastuzumab-based therapy, has been reported in 13% of patients receiving paclitaxel and trastuzumab (including severe events in 2% of patients) (Slamon *et al*, 2001). In the study by Marty *et al* (2005), one patient (1%) receiving trastuzumab and docetaxel experienced symptomatic heart failure, and another patient experienced symptomatic heart failure 5 months after discontinuation of trastuzumab while receiving an investigational anthracycline for 4 months. The favourable safety profile of the vinorelbine/trastuzumab combination has been observed in other trials: Burstein *et al* (2003) reported only one patient with grade 3 cardiac toxicity, whereas no grade 3–4 cardiac toxicity was seen by Jahanzeb *et al* (2002).

It is important to note that cardiac toxicity was reported in 4.7% of patients with trastuzumab monotherapy (Cobleigh *et al*, 1999). The 5.9% incidence of cardiac toxicity seen in the present study with only one patient (1.5%) experiencing symptomatic cardiac failure is therefore similar to that seen when trastuzumab is used alone, reflecting the absence of additional cardiac toxicity from vinorelbine. Twenty per cent of patients were treated for more than 1 year.

In conclusion, the vinorelbine/trastuzumab regimen is an effective and well-tolerated first-line therapy option for HER 2-overexpressing metastatic breast cancer patients. The shorter duration of administration without the need of a steroid premedication and lack of cumulative side effects with marginal cardiac toxicity provide strong argument for its choice as the control arm in future phase III trials including trastuzumab-based combinations.

## Figures and Tables

**Table 1:** Patient's characteristics

**Characteristic**	***N***	**(%)**
Included	69	
		
*Age (years)*		
Median	53	
Range	30–74	
		
KPS ⩾70%	69	100
		
*HER 2 testing*		
IHC 3+	67	97.1
IHC 2+/FISH+	2	2.9
		
Prior hormonotherapy	34	49.3
		
*Prior chemotherapy*	45	65.2
Neoadjuvant	5	7.2
Adjuvant	30	43.5
Both	10	14.5
		
*Chemotherapy regimen*		
Anthracycline based	23	51.1
Anthracycline+taxane	13	28.9
CMF	9	20.0
		
*Metastatic sites ⩾2*	22	31.9
Lymph nodes	35	50.7
Visceral (lung/liver)	52	75.4
Liver	38	55.1
Lung	20	29.0
Bone	31	44.9
		
*Disease-free interval from diagnosis to first relapse (months)*		
Median	17.8	
Range	0–202.7	

CMF=cyclophosphamide methotrexate 5-fluorouracil; FISH=fluorescent *in situ* hybridisation; IHC=immunohistochemistry; KPS=Karnofsky performance status.

**Table 2:** Antitumour efficacy

**Best overall response (Independent Review Panel)**	***N***	**(%)**
Evaluable patients	62	
CR	9	14.5
PR	30	48.4
Objective response (CR+PR) (95% CI)	39	62.9 (49.7–74.8)
SD	12	19.4
Clinical benefit (CR+PR+SD ⩾6 months) (95% CI)	45	72.6 (61.5–83.7)
PD	11	17.7
		
*Prognostic factors of response rate (patients)*		
Prior chemotherapy regimen		
No prior adjuvant/neoadjuvant (23)	16	69.6
Anthracycline based (21)	13	61.9
Anthracycline+taxane (11)	6	54.5
Visceral involvement (liver/lung)		
Yes (47)	30	63.8
No (15)	9	60.0
Stage IV at diagnosis (12)	9	75.0

CI=confidence interval; CR=complete response; PD=progressive disease; PR=partial response; SD=stable disease.

**Table 3:** Survival data (cutoff date 1 October, 2004)

Median time to response (weeks) (range)	8.4 (7.1–31.3)
Median duration of response (months) (95% CI)	17.5 (12.1–23.0)
Median progression-free survival (months) (95% CI)	9.9 (5.6–12.1)
Median survival (months) (95% CI)	23.7 (18.4–32.6)

CI=confidence interval.

**Table 4:** Worst haematological WHO grades, by evaluable patient (*N*=68^a^) and by evaluable administration of vinorelbine+trastuzumab (*N*=1705)

		**Patients**		**Administrations**	
**Haematological toxicity**	**Patients (overall incidence)**	**Grade 3, *N* (%)**	**Grade 4, *N* (%)**	**Grade 3, *N* (%)**	**Grade 4, *N* (%)**
Anaemia	58 (85.3)	2 (2.9)	—	3 (0.2)	—
Leucopenia	63 (92.6)	39 (57.4)	6 (8.8)	159 (9.3)	13 (0.8)
Neutropenia	63 (92.6)	28 (41.2)	29 (42.6)	226 (13.3)	75 (4.4)
Febrile neutropenia^b^	2 (2.9)	—	—	—	—
Thrombocytopenia	4 (5.9)	—	—	—	—

WHO=World Health Organisation.

a One patient with only one treatment administration was not evaluable for haematological toxicity.

b One of these patients died of neutropenic sepsis.

**Table 5:** Worst related nonhaematological WHO grades, by evaluable patient (*N*=69) and by evaluable cycle (*N*=613) of vinorelbine+trastuzumab

		**Patients**		**Cycles**	
**Nonhaematological toxicity**	**Patients (overall incidence)**	**Grade 3, *N* (%)**	**Grade 4, *N* (%)**	**Grade 3, *N* (%)**	**Grade 4, *N* (%)**
Anorexia	18 (26.1)	2 (2.9)	—	3 (0.5)	—
Nausea/vomiting	42 (60.9)	1 (1.4)	—	1 (0.2)	—
Constipation	20 (29.0)	2 (2.9)	—	2 (0.3)	—
Diarrhoea	18 (26.1)	1 (1.4)	1 (1.4)	1 (0.2)	1 (0.2)
Stomatitis	27 (39.1)	—	—	—	—
Peripheral neuropathy	22 (31.9)	2 (2.9)	—	3 (0.5)	—
Asthenia	33 (47.8)	6 (8.7)	—	10 (1.6)	—
Chills	15 (21.7)	1 (1.4)	—	1 (0.2)	—
Hair loss	25 (36.2)	—	—	—	—
Cutaneous	14 (20.3)	1 (1.4)	—	1 (0.2)	—
Drug fever	23 (33.3)	1 (1.4)	—	1 (0.2)	—
Infection	18 (26.1)	4 (5.8)	—	5 (0.9)	—
Local venous toxicity	16 (23.2)	1 (1.4)	—	2 (0.3)	—

WHO=World Health Organisation.
